# Enhancement of Optical Adaptive Sensing by Using a Dual-Stage Seesaw-Swivel Actuator with a Tunable Vibration Absorber

**DOI:** 10.3390/s110504808

**Published:** 2011-05-03

**Authors:** Po-Chien Chou, Yu-Cheng Lin, Stone Cheng

**Affiliations:** Department of Mechanical Engineering, National Chiao Tung University, No. 1001, University Road, Hsinchu 30010, Taiwan; E-Mails: arthurking8@msn.com (P.-C.C.); owan0518@hotmail.com (Y.-C.L.)

**Keywords:** small-form-factor, VCM, PZT, swing arm, absorber

## Abstract

Technological obstacles to the use of rotary-type swing arm actuators to actuate optical pickup modules in small-form-factor (SFF) disk drives stem from a hinge’s skewed actuation, subsequently inducing off-axis aberrations and deteriorating optical quality. This work describes a dual-stage seesaw-swivel actuator for optical pickup actuation. A triple-layered bimorph bender made of piezoelectric materials (PZTs) is connected to the suspension of the pickup head, while the tunable vibration absorber (TVA) unit is mounted on the seesaw swing arm to offer a balanced force to reduce vibrations in a focusing direction. Both PZT and TVA are designed to satisfy stable focusing operation operational requirements and compensate for the tilt angle or deformation of a disc. Finally, simulation results verify the performance of the dual-stage seesaw-swivel actuator, along with experimental procedures and parametric design optimization confirming the effectiveness of the proposed system.

## 1. Introduction

Swing arm actuators have been applied to small form factor (SFF) optical pickup heads or near field hybrid recording systems. Trends in optical disk drives include developing small-form-factor (SFF) for holographic optics and extending the applications of near-field optics. In such a high speed positioning servo system, the head/disk spacing variation must be maintained as small as possible during disk operations. While attempting to reduce the size of optical heads, several works have scaled down all components of a conventional unit [1,2] and simplified the optical configuration, and micro optical elements, such as lenses and prisms. Other approaches integrate an optical module with a seesaw swivel actuator [3,4].

Two possible actuated mechanisms are available for active and passive head-positioning control. For active actuation, Yeack-Scranton devised a secondary stage PZT bender with an active slider for contact recording [5]. Following the PZT actuator and its applications, Tagawa proposed a self-loading slider featuring a twin structure piezo-actuator to maintain constant flying height spacing [6]. Kajitani developed an active slider associated with multilayered piezo-actuators to compensate for flying height variation [7]. In contrast with the above track-seek operations, Jun and Takashi investigated the feasibility of using a circular arc slider driven by a piezoelectric actuator to reduce the tilt motions of the lens in pitching/rolling directions [8]. Liu investigated the feasibility of a dynamic motion approach for a piezotube actuator subjected to disk deformation [9]. Moreover, in study involving a multilayered structure, Woosung used a flexure hinge mechanism to extend the allowable stroke to tracking and focusing motions [10]. Additionally, passive mass absorber utilizes an inertial mass and tuned support to mitigate mechanical vibration. Chung introduced a two-dimensional vibration model of a feeding deck system to reduce in-plane vibration [11]. According to their functional and structural identification, Heo characterized the dynamic behavior of a passive damper consisting of a rubber bobbin and plate ring [12]. Moreover, Chung established a design procedure to combine passive and active devices with an enlarged bandwidth control. That adaptive dynamic absorber is embedded with a voice coil motor (VCM) device [13]. However, to make use of the hollow space within the VCM, Jiang investigated the feasibility of a tuned dual mass damping device [14]. In particular, Kuwajima developed a novel shockproof structure by using a balanced type suspension, as well as investigated its performance on external shock resistance during read/write operations [15].

**Figure 1.**
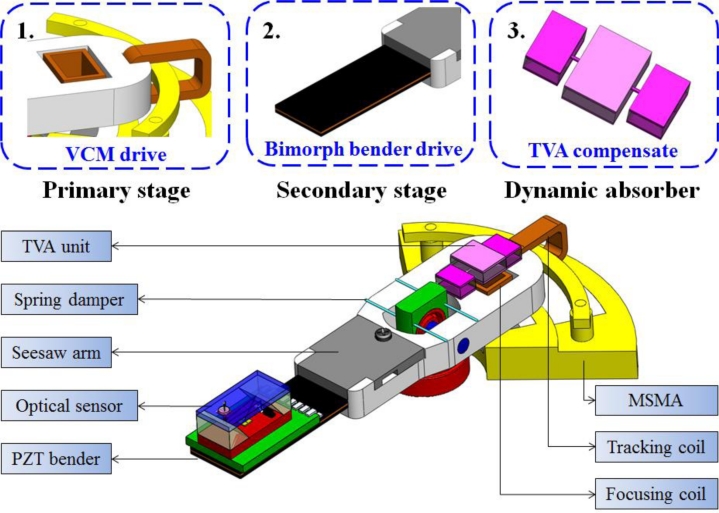
Schematic illustration of the bimorph dual-stage actuator with the TVA unit indicated.

This work describes a novel a dual-stage seesaw-swivel actuator based on optical sensing by using a PZT bimorph component to compensate for the tilt angle variation in focusing stroke. A piezoelectric actuated suspension for a secondary-stage actuator is also developed to implement fine head positioning to assist the primary VCM actuator and maintain a constant flying height during the focusing operation, as shown in Figure 1. The secondary micro actuator functions mainly for fine and fast compensation, while a conventional VCM performs coarse positioning. A dynamic model for a bimorph structure is also developed to optimize and verify the natural frequency of actuating behavior. Additionally, a two dimensional vibration model of the optical sensing device with the absorber is established to ensure system stability and robustness. Based on multi-body dynamic analysis, a balanced mass associated with two cantilever springs provides a rotational reaction to the moving part under seesaw actuation. Moreover, this work investigates the feasibility of implementing a passive tunable vibration absorber (TVA) for spacing variation and head positioning control endowed with a bimorph micro actuator. Finally performance of biaxial motion is evaluated based on numerical simulations of optimization and experimental results.

## 2. Mechanical Design and Theoretical Analysis

The optical axis shift during the primary VCM actuation is compensated for by using the active bimorph micro actuator. A TVA can passively absorb the residual tilt variations while actively generating a reacting force for the moment conservation. The proposed mechanism is a seesaw type swivel actuator using piezoelectric active control collaboratively with passive TVA design. The following sections describe the proposed mechanism.

### 2.1. Angular Deviation Compensation

**Figure 2.**
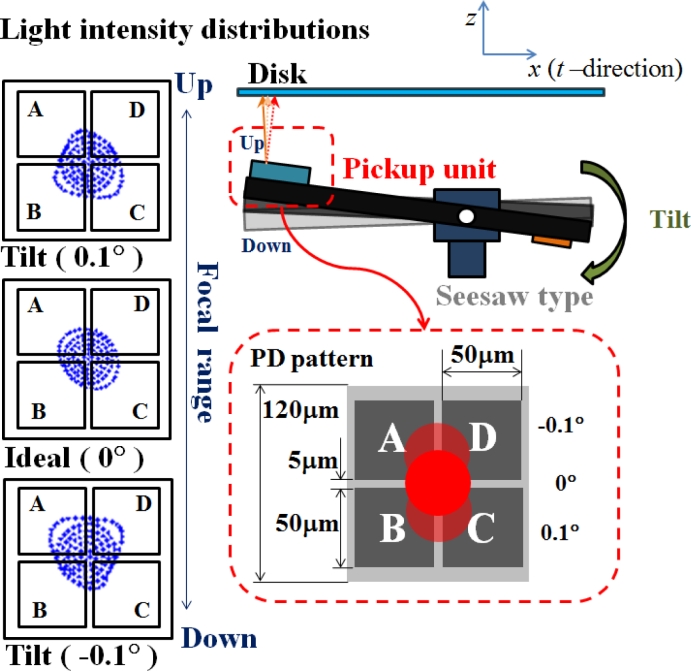
Misalignment in the incline angle of the optical axis.

In actuated slider suspension, the tilt motion of the optical actuator causes the variation of focal spot position on the photo-detector, as shown in Figure 2. The middle figure of light intensity distribution is a well-symmetric focal spot on the PD center without tilting angle. If the focusing motion is achieved by z seesaw-type arm which generates a ±0.1° nutating angle, the returning beam would not be orthogonal to the optical sensor. The reflected focal spot on the PD will shift away from the center and the light distributions will approach one side of the photo-diode. Hence, given the increasing accuracy of head positioning, a drive capable of tilt control is necessary to reduce the tilt margin as the optical aberration is correspondingly increased. A compensation scheme is also developed for optical axis in-line calibration, and evaluated by driving a simply bimorph PZT bender.

### 2.2. Dual-Stage Leveraged Mechanism

**Figure 3.**
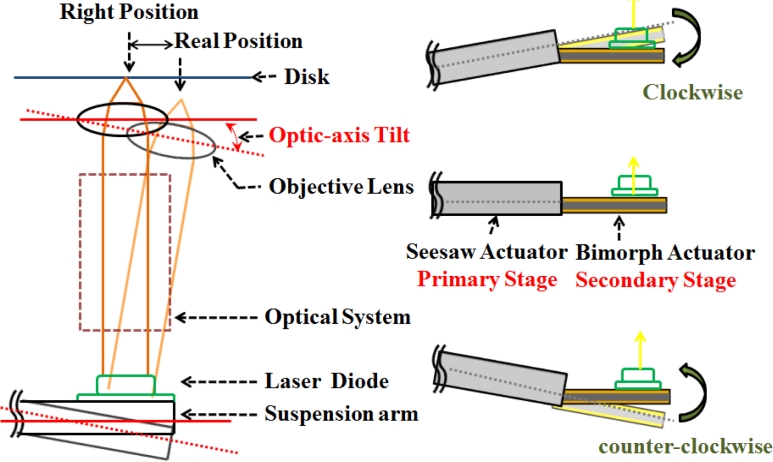
Conceptual diagram of the combined bimorph piezo-VCM actuator based active tilt compensation.

A common method of piezoelectric actuated suspension is driven along the opposite data (track) tangential direction in an allowable stroke. The proposed seesaw swivel-drive actuator uses a dual-stage leverage mechanism (Figure 3), through use of a coarse movement via VCM control and a secondary fine positioning via PZT. As PZT compensates the tilt angle of seesaw arm in the opposite way—the laser beam will be kept in orthogonal to the disk (Figure 3, left). Hence, PZT tilt control fixes the tangential displacement of focusing, and calibrates the asymmetry of the S-curve. Complete two-axis scanning is then performed by independently controlling the dual stage actuators.

### 2.3. Cantilever TVA Structure

The primary structure with arbitrary distributions of mass, stiffness and damping is subjected to vibration suppression by an absorber subsystem. Sensing variation capabilities in the optic-axis direction are investigated to ensure the suppression of robust vibration. Consisting of two flexible cantilever beams extended from a balanced mass, a cantilever TVA subsystem is illustrated from the perspective of both improving bandwidth and increasing shock protection.

**Figure 4.**
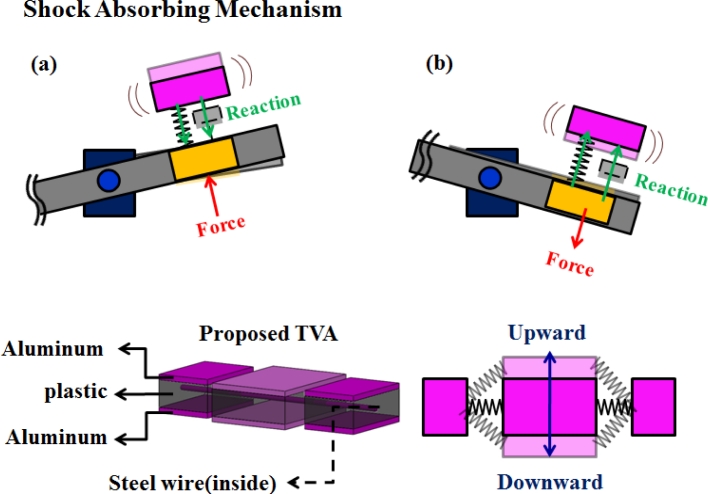
Developed tunable vibration absorber with system dynamics behavior: principles and practices.

The vibration suppression of structures is based on independent design of dynamic absorbers, while taking into account the selected tuning mass of the subsystem structure. Effective structural stiffness is thus adjusted by adjusting the beam dimensions after a preliminary primitive design. Figure 4 illustrates the structural configuration of this work, which consists of a three-block structure called “soundmetal” that is pinned by a flexible wire.

## 3. Numerical Analysis

DeVoe and Pisano [16] developed a model for piezoelectric multi-morph cantilevers extended from Timoshenko’s approach to investigate the deflection of micro-actuators. To derive a simple working equation, Weinberg [17] obtained a simple closed-form solution for the bending of piezoelectric multi-morphs by using the Euler-Bernoulli beam theory and integrated equilibrium equations. This theory assumes that cross sections remain plane and normal to the deformed beam axis. Such assumptions are appropriate given the slender geometry of typical multi-morphs. Krommer developed the constitutive equations of a multi-morph by assuming two-dimensional kinematics of the structure [18]. Ha and Kim attempted to optimize bimorphs in order to maximize the tip stroke or vertical displacement by using the thickness ratio between the substrate (or shim layer) and PZT layers [19]. Pursuing a high bandwidth and a sufficient optical performance of the combined piezo-VCM actuator and design parameters of the PZT bender (*i.e.*, length, width and thickness) are of priority concern. Generally, the rotary angle and deflection of the PZT bender is proportion to the square and cube of the length of the PZT bender (by basic beam theory). Other parameters affect the bandwidth (fundamental frequency).

### 3.1. Rotary Angle of PZT Bender

**Figure 5.**
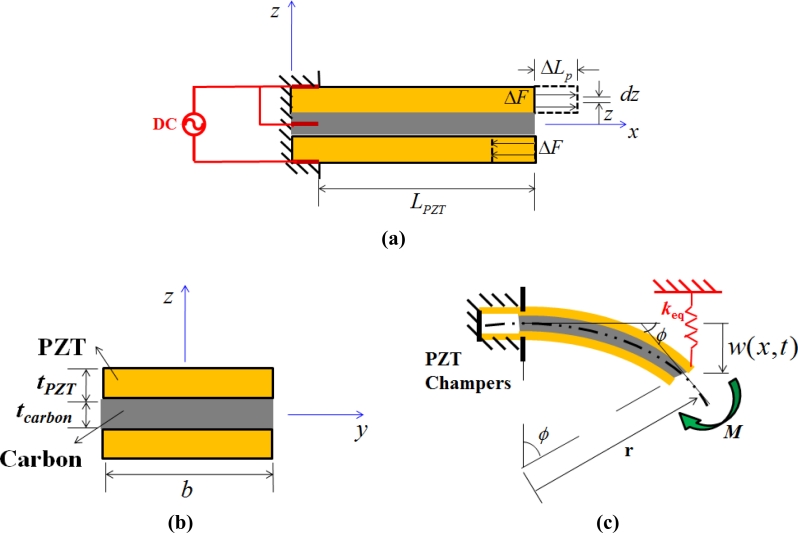
Structure variables of triple-layer bimorph actuator: **(a)** crosswise view; **(b)** cross-section view; **(c)** deflection skew and equivalent spring.

Figure 5 displays a “sandwich”-like PZT that consists of a carbon fiber chip with two *d_31_*-type PZT ceramics. This figure also considers a small piece element in the upper PZT material, in which the corresponding extension can be evaluated as follows:

(1) $$\frac{\Delta L_{\text{PZT}}}{L_{\text{PZT}}}\;=\;\frac{d_{31}V_{\text{PZT}}}{t_{\text{PZT}}}$$

The elongation force *ΔF* and momentum *ΔM* generated by the small element in the Figure 4(a) are:

(2) $$\Delta M\;=\;\Delta F\;\cdot \;z\;=\;\frac{d_{31}V_{\text{PZ}T}bE_{\text{PZT}}}{t_{\text{PZT}}}\text{zdz}$$

Therefore, integrating Equation (2), the sum of bending moment in Figure 4 generated by the bimorph piezoelectric material is as follows:

(3) $$M\;=\;d_{31}\cdot V_{\text{PZT}}\;\cdot \;E_{\text{PZT}}\;\cdot \;b\;\cdot \;(t_{\text{PZT}}\;+\;t_{\text{carbon}})$$

Figure 5(a) shows a composed triple-layer beam that consists of both carbon fiber and piezoelectric material. The flexural rigidity of a multilayered beam is:

(4) $$E_{\text{eq}}\;I_{\text{eq}}\;=\;E_{\text{PZT}}I_{\text{PZT}}\;+\;E_{\text{carbon}}\;I_{\text{carbon}}$$

where *k_PZT_* is considered the stiffness in rotation direction, and calculated from the equivalent spring on the tip of PZT bender.

(5) $$k_{\text{PZT}}\;=\;k_{\text{eq}}\;\frac{L_{\text{PZT}}}{2}$$

where *k_eq_* in Figure 5(c) is presented as equivalent spring constant.

(6) $$k_{\text{eq}}\;=\;\frac{2EI}{L_{\text{PZT}}^{2}}$$

Additionally, the rotary angle of PZT is:

(7) $$\phi \;=\;\frac{M}{k_{\text{PZT}}}$$

### 3.2. Natural Frequency of PZT Bender

The motion of a cantilever beam can be represented as [16]:

(8) $$c^{2}\;\frac{\partial^{4}\;w(x,\;t)}{\partial x^{4}}\;+\;\frac{\partial^{2}\;w(x,\;t)}{\partial t^{2}}\;=\;0,\;c\;=\;\sqrt{\frac{E_{eq}I_{eq}}{m\prime }}$$

By using the separation of variables method, the general solution can be expressed as

(9) w(x, t) = X (x) ⋅ T(t)

Replacing Equation (8) with Equation (9) yields

(10) $$c^{2}\;\frac{X\prime \prime \prime \prime \;(x)}{X\;(x)}\;=\;-\frac{T\prime \prime (t)}{T(t)}\;=\;\omega_{n}^{2}$$

The general solution of the mode shapes in *x*-direction can be expressed as

(11) $$X_{n}\;(x)\;=\;A_{1}\;\text{sin}\;(\lambda_{n}x)\;+\;A_{2}\;\text{cos}\;(\lambda_{n}x)\;+\;A_{3}\;\text{sinh}\;(\lambda_{n}x)\;+\;A_{4}\;\text{cosh}\;(\lambda_{n}x),\;\lambda_{n}^{4}\;=\;\frac{m^{\prime }\omega_{n}^{2}}{E_{\text{eq}}I_{\text{eq}}}$$

The boundary condition of Equation (10) is:

(12) $$\{\begin{array}{l} x\;=\;0\;\Rightarrow \;w\;=\;0,\;\frac{\partial w}{\partial x}\;=\;0\\ x\;=\;L_{\text{PZT}}\;\Rightarrow \;EI\frac{\partial^{2}w}{\partial x^{2}}\;=\;M\;=\;0,\;EI\frac{\partial^{3}w}{\partial x^{3}}\;=\;V\;=\;0 \end{array}$$

By substituting Equation (12) into Equation (11), the particular solution is

(13) $$X_{n}(x)\;=\;\text{cosh}\left(\frac{\lambda_{n}x}{L_{\text{PZT}}}\right)-\text{cos}\left(\frac{\lambda_{n}x}{L_{\text{PZT}}}\right)-\;\alpha \left(\text{sinh}\left(\frac{\lambda_{n}x}{L_{\text{PZT}}}\right)-\text{sin}\left(\frac{\lambda_{n}x}{L_{\text{PZT}}}\right)\right)$$

where $\alpha \;=\;\frac{\text{sinh}\;(\lambda_{n}L_{\text{PZT}})\;-\;\text{sin}\;(\lambda_{n}L_{\text{PZT}})}{\text{cosh}\;(\lambda_{n}\;L_{\text{PZT}})\;+\;\text{cos}\;(\lambda_{n}L_{\text{PZT}})}$

The natural frequency is solved from the following equation:

(14) $$\text{cos}(\lambda_{n}x)\;\text{cosh}(\lambda_{n}x)\;=\;-1;\;n=1,\;2,\;3,\ldots$$

For *n* = 1, the fundamental frequency of the bimorph PZT is:

(15) $$\omega_{1}\;=\;\sqrt{\frac{E_{eq}I_{eq}}{m\prime }}\cdot \frac{1.875^{2}}{2\pi L_{\text{PZT}}^{2}}\;(Hz)$$

where *m′* = [*ρ_PZT_t_PZT_* + *ρ_carbon_t_carbon_*]*b*.

### 3.3. Optimization

**Figure 6.**
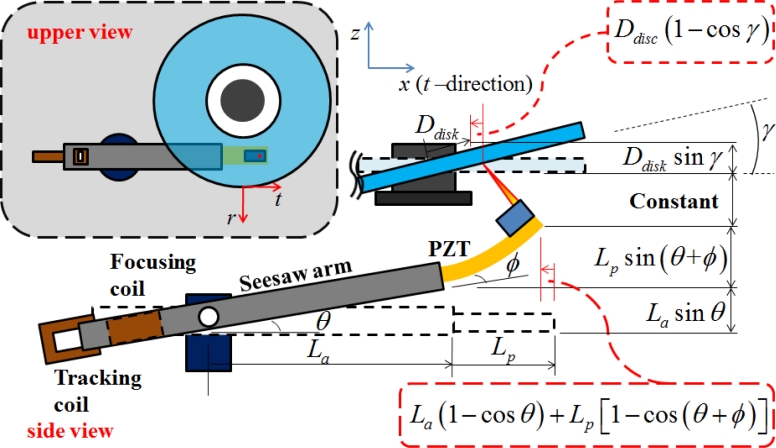
Geometry and coordinates for the dual stage actuator based on electromagnetism and mechanism (*t* and *r* present tangential and radius direction respected to the disk).

From the previous derivations of the equations, two objective functions of the optimization are selected: lateral displacement error *E*(*t_PZT_*, *L_PZT_*) and fundamental frequency *ω*(*t_PZT_*, *L_PZT_*). Figure 6 shows the relationship between PZT arm actuator and disk, while Table 1 lists its symbols.

**Figure 7.**
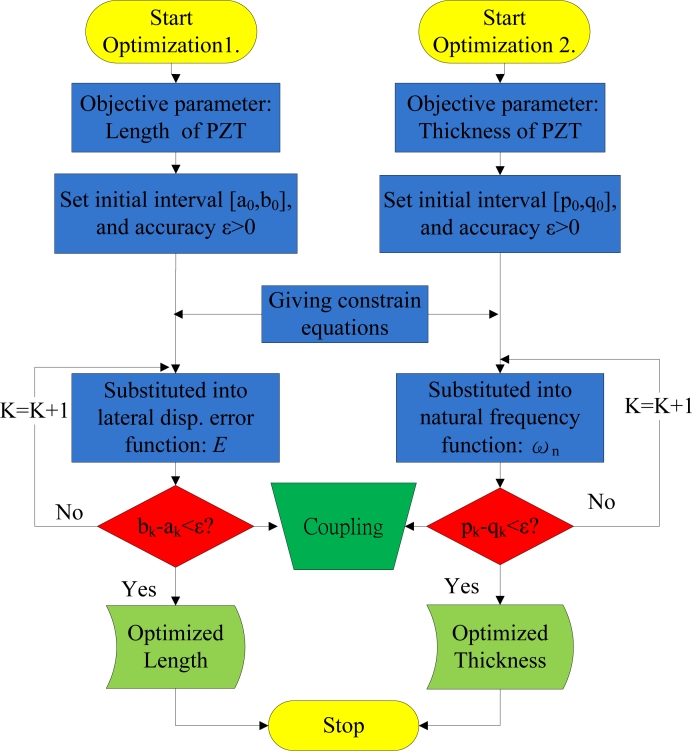
Flowchart of the PZT optimization procedure.

Figure 7 illustrates the optimization procedure, indicating that the PZT bender is optimized. Of the two coupling optimization processes, optimization 1 determines objective functions and sets the initial search interval and accuracy. Meanwhile, optimization 2 copes with constraint conditions for the process. The optimization of length *L_PZT_* and thickness *t_PZT_*, which minimize objective functions to PZT, are 14.13 mm and 0.11 mm after 64 and 59 iterations, respectively.

**Figure 8.**
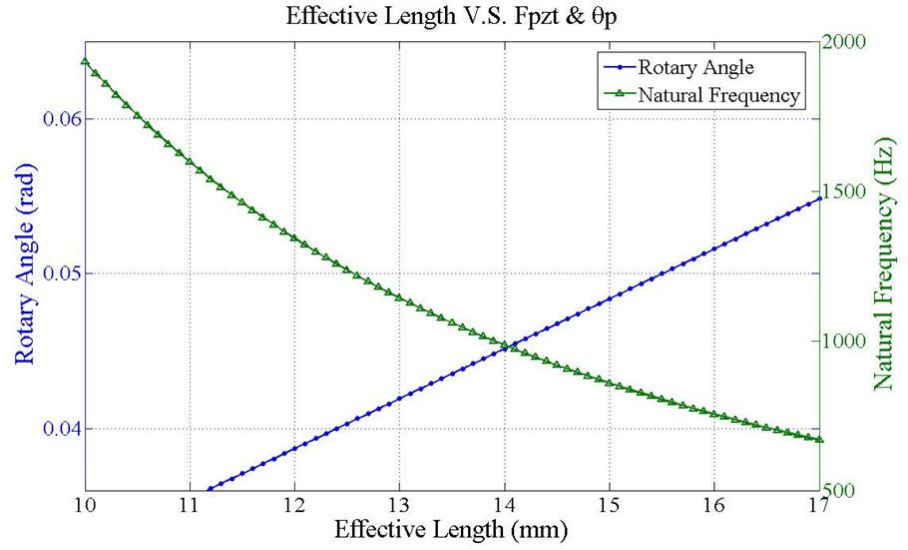
Effective length *versus* optimal balance between rotary angle (rad) and natural frequency (Hz).

**Figure 9.**
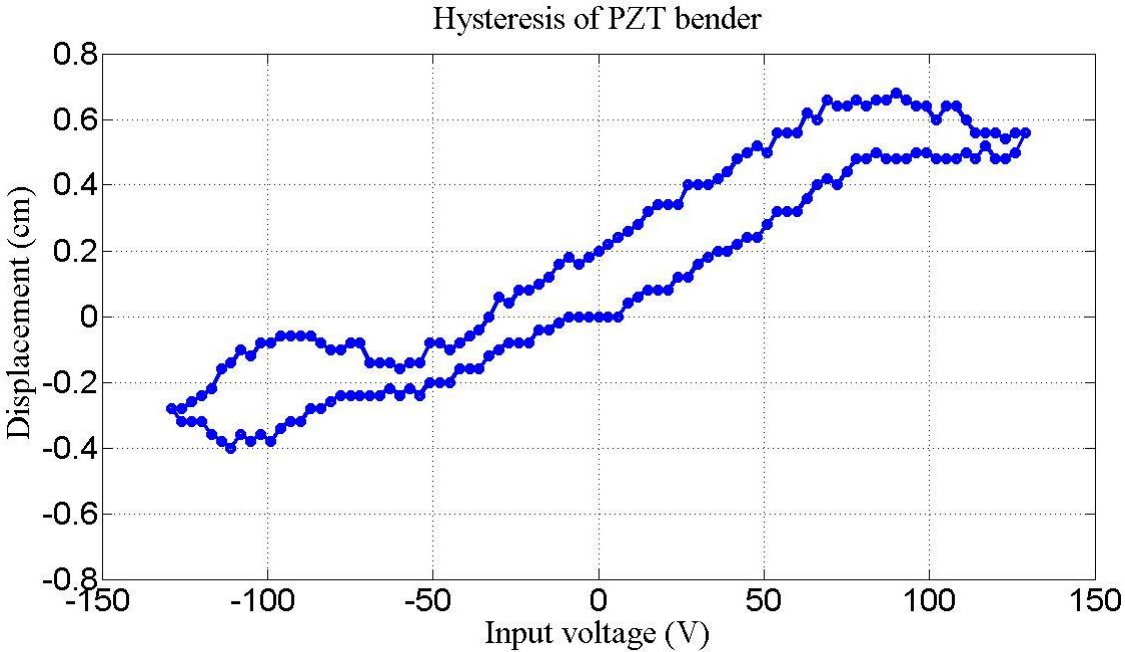
Deflection curve of hysteresis loop in a bimorph bender with the applied voltage swing from ±125 V.

**Figure 10.**
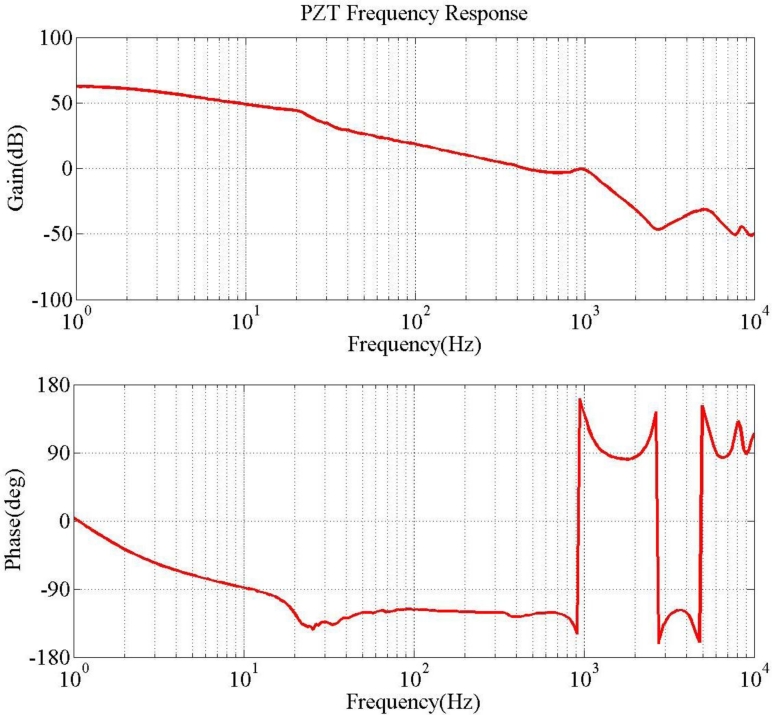
Measured frequency response of optimum PZT bender.

Figure 8 plots two parameters, rotary angle ϕ [Equation (7)] and fundamental frequency ω_1_ [Equation (15)] as a function of effective length. The crossover point is selected to verify the optimization results. Figure 9 illustrates the hysteresis curve of PZT displacement with maximum point is 4 mm when input a sine wave signal is swept from −125 to 125 V. Finally, the experimental results in Figure 10 demonstrate two modes frequency at 985 Hz and 5.8 kHz. Table 2 summarizes the modal analysis results between the numerical optimization and experiments, in which the errors vary by about 1.86% and 3.14%, respectively.

### 3.4. Absorber Mass Tuning

Several attempts have been made to seek optimum absorber parameters when the main system has significant damping. Den Hartog first tackled the optimum solution of a tuned mass damper that is connected to a primary system with the intention of reducing its dynamic response [20]. An optimal broadband attenuation was then achieved by a dynamic absorber design proposed by Ormondroyd and Den Hartog [21]. Based on their fixed-point theory, Brock found the optimum tuning parameter and defined the optimality for the optimum absorber damping [22]. By adopting a frequency locus approach, Thompson found the optimal damper parameters that minimize the structural response [23].

**Figure 11.**
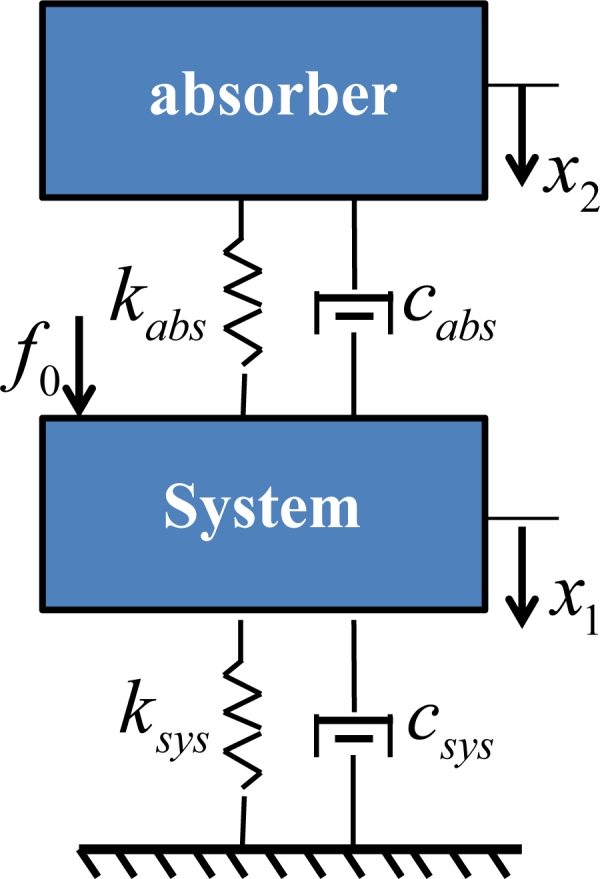
A suspended mass *m_sys_* interfered with a driven force *f_0_*. The absorber mass *m_abs_* is maneuvered by the reaction force to reduce displacement *x_1_*.

The equation of the damped model in Figure 11 can be expressed as

(16) $$\{\begin{array}{ccc} m_{\text{sys}}{\ddot{x}}_{1}\;-\;c_{\text{abs}}\;({\dot{x}}_{2}\;-\;{\dot{x}}_{1})\;-\;k_{\text{abs}}\;(x_{2}\;-\;x_{1})\;+\;c_{\text{sys}}{\dot{x}}_{1}\;+\;k_{\text{sys}}x_{1} & = & f_{0}\\ m_{\text{abs}}{\ddot{x}}_{2}\;+\;c_{\text{abs}}\;({\dot{x}}_{2}\;-\;{\dot{x}}_{1})\;+\;k_{\text{abs}}\;(x_{2}\;-\;x_{1}) & = & 0 \end{array}$$

where *m_sys_, c_sys_* and *k_sys_* denote the mass, damping and stiffness coefficient of primary actuator system; *m_abs_, c_abs_,* and *k_abs_* refer to the mass, damping, and stiffness coefficient of tunable vibration absorber system; *f_0_* and *ω* denote the amplitude and frequency of the exciting force, respectively; and *x_1_* and *x_2_* represent the displacement of the actuator and the absorber, respectively. The normalized amplitude of the steady-state response of the primary mass can be expressed as:

(17) $$\{\begin{array}{ccc} x_{1} & = & X_{1}e^{j\omega t}\\ x_{2} & = & X_{2}e^{j\omega t}\\ f_{0} & = & F_{o}e^{j\omega t} \end{array}$$

The amplitudes of the system displacement, *X_1_*, are expressed as:

(18) $$X_{1}\;=\;\frac{\left(k_{\text{abs}}\;-\;m_{\text{abs}}\omega^{2}\right)\;+\;j\omega c_{\text{abs}}}{\left\{\begin{array}{l} \left(k_{\text{sys}}\;-\;m_{\text{sys}}\omega^{2}\right)\left(k_{\text{abs}}\;-\;m_{\text{abs}}\omega^{2}\right)\;-\;\left(k_{\text{abs}}m_{\text{abs}}\;+\;c_{\text{sys}}c_{\text{abs}}\right)\omega^{2}\\ +\;j\omega \left[c_{\text{abs}}\;\left(k_{\text{sys}}\;-\;m_{\text{sys}}\;\omega^{2}\;-\;m_{\text{abs}}\omega^{2}\right)\;+\;c_{\text{sys}}\;\left(k_{\text{abs}}\;-\;m_{\text{abs}}\omega^{2}\right)\right] \end{array}\right\}}\cdot \;F_{0}$$

Taking the absolute of *X*_1_:

(19) $$|X_{1}|\;=\;F_{0}\sqrt{\frac{{\left[{\left(\frac{k_{a}}{m_{a}}-\omega^{2}\right)}^{2}\;+\;\left(\frac{C_{a}}{m_{a}}\omega \right)\right]}^{2}\;{\left(\frac{k_{\text{sys}}}{m_{\text{sys}}}\right)}^{2}}{{\left[\left(\frac{k_{\text{sys}}}{m_{\text{sys}}}-\omega^{2}\right)\left(\frac{k_{a}}{m_{a}}-\omega^{2}\right)-\left(\frac{k_{a}}{m_{a}}\frac{m_{a}}{m_{\text{sys}}}+\frac{C_{a}}{m_{a}}\frac{C_{\text{sys}}}{m_{\text{sys}}}\right)\omega^{2}\right]}^{2}\;+\;{\left[\frac{C_{a}}{m_{a}}\left(\frac{k_{\text{sys}}}{m_{\text{sys}}}-\omega^{2}-\frac{m_{a}}{m_{\text{sys}}}\omega^{2}\right)\;+\;\frac{C_{\text{sys}}}{m_{\text{sys}}}\left(\frac{k_{a}}{m_{a}}-\omega^{2}\right)\right]}^{2}}}$$

Substituting the natural frequency, $\omega_{\text{abs}}\;=\;\sqrt{\frac{k_{\text{abs}}}{m_{\text{abs}}}}$ and $\omega_{\text{sys}}\;=\;\sqrt{\frac{k_{\text{sys}}}{m_{\text{sys}}}}$, into the Equation (19), the normalized transfer function of an actuator is

(20) $$G\;=\;\frac{\left|X_{1}\;\cdot \;k_{\text{sys}}\right|}{\left|f_{0}\right|}=\left|X_{1}\right|\cdot \frac{k_{\text{sys}}}{F_{0}}\;=\;\sqrt{\frac{A^{2}\;(\alpha ,\;\beta )\;+\;4B^{2}\;(\zeta_{\text{abs}},\;\alpha ,\;\beta )}{C^{2}(\mu ,\;\zeta_{\text{sys}},\;\zeta_{\text{abs}},\;\alpha ,\;\beta )\;+\;4D^{2}\;(\mu ,\;\zeta_{\text{sys}},\;\zeta_{\text{abs}},\;\alpha ,\;\beta )}}$$

where:

$$\begin{array}{l} A(\alpha ,\;\beta )\;=\;1-\frac{\alpha^{2}}{\beta^{2}},\;B(\zeta_{\text{abs}},\;\alpha ,\;\beta )\;=\;\zeta_{\text{abs}}\frac{\alpha }{\beta }\\ C(\mu ,\;\zeta_{\text{sys}},\;\zeta_{\text{abs}},\;\alpha ,\;\beta )\;=\;\frac{\alpha^{4}}{\beta^{2}}-\left(\frac{4\zeta_{\text{sys}}\zeta_{\text{abs}}}{\beta }\;+\;\frac{1}{\beta^{2}}+\mu +1\right)\alpha^{2}\;+\;1\\ D(\mu ,\;\zeta_{\text{sys}},\;\zeta_{\text{abs}},\;\alpha ,\;\beta )\;=\;\zeta_{\text{abs}}\;\left(\frac{\alpha }{\beta }-\frac{\alpha^{3}}{\beta }\;-\;\mu \frac{\alpha^{3}}{\beta }\right)\alpha^{2}\;+\;\zeta_{\text{sys}}\;\left(\alpha \;-\;\frac{\alpha^{3}}{\beta^{2}}\right) \end{array}$$

and the parameters are:

$$\mu \;=\;\frac{m_{\text{abs}}}{m_{\text{sys}}},\;\alpha \;=\;\frac{\omega }{\omega_{\text{sys}}},\;\xi_{\text{sys}}\;=\;\frac{C_{\text{sys}}}{2\sqrt{m_{\text{sys}}k_{\text{sys}}}},\;\beta \;=\;\frac{\omega_{\text{abs}}}{\omega_{\text{sys}}},\;\xi_{\text{abs}}\;=\;\frac{C}{2\sqrt{m_{\text{abs}}k_{\text{abs}}}}$$

According to Den Hartog’s theorem [17], the ratio of natural frequency between absorber and system is:

(21) $$\beta^{*}\;=\;\frac{1}{1\;+\;\mu }$$

**Figure 12.**
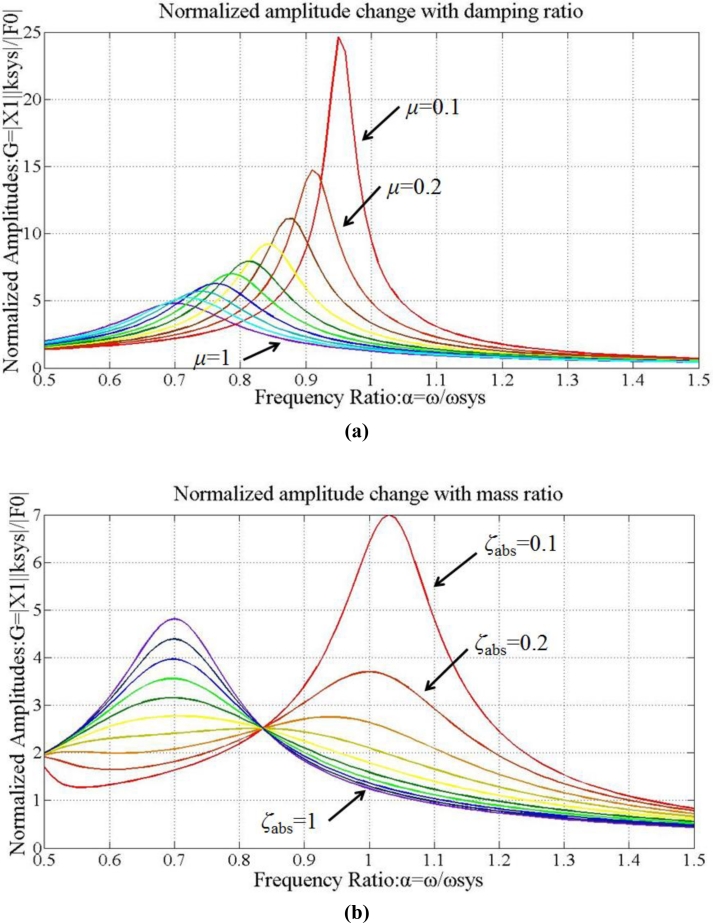
Normalized amplitudes of vibrations: **(a)** *ζ_abs_* = 1 (fixed), *μ* = 0.1∼1. **(b)** *μ* = 1 (fixed), *ζ_abs_* = 0.1∼1.

Figure 12(a) displays the relationship between *μ* and *G*. Hence, the peak is reduced with enlarged *μ*. According to Figure 12(b), the resonance peak frequency increases when *ζ_abs_* decreases from 1 down to 0.1. The optimal damping ratio of the absorber, $\zeta_{\text{abs}}^{*}$, is estimated as:

(22) $$\zeta_{\text{abs}}\;=\;\zeta_{\text{abs}}^{*}\;=\;\sqrt{\frac{3\mu }{8(1\;+\;\mu )}}$$

**Figure 13.**
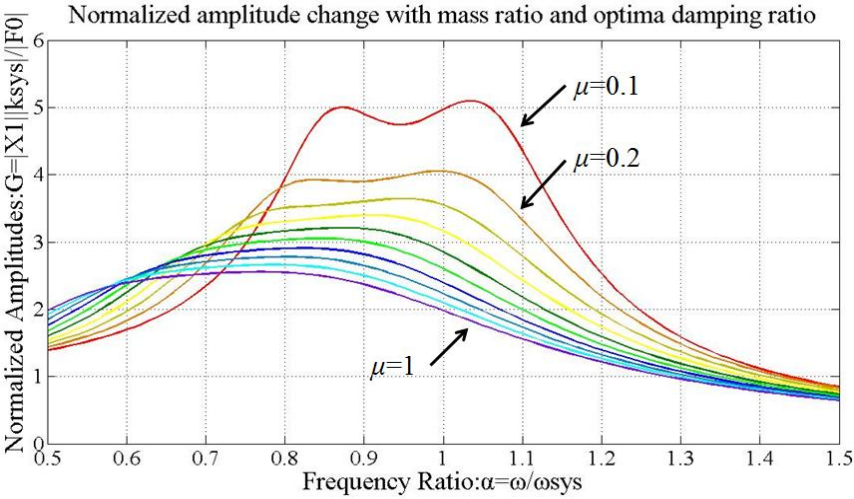
Normalized amplitudes of vibrations when *μ* = 0.1∼1 and *ζ_abs_* = $\zeta_{\text{abs}}^{*}$.

Figure 13 shows the normalized amplitude *G* as a function of *μ*. By assigning the optimal damping ratio *ζ_abs_* and mass ratio *μ*, the vibration magnitudes are decreased effectively. (Therefore, the actual values of mass and stiffness of TVA are determined by selecting *μ* = 0.8 and $\zeta_{\text{abs}}^{*}$ = 0.408.

## 4. Experiments

### 4.1. Evaluation of Dynamic Performance

**Figure 14.**
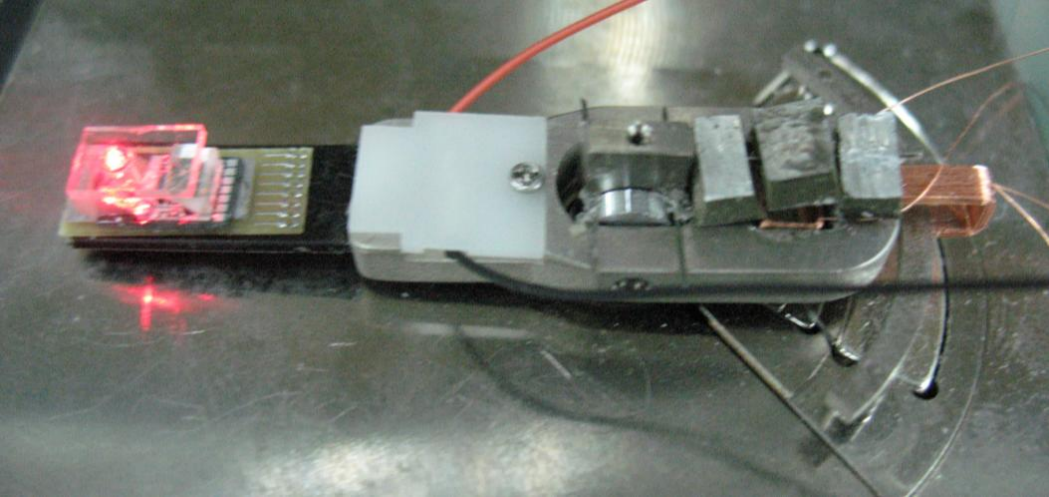
Prototype of the dual-stage seesaw actuator.

**Figure 15.**
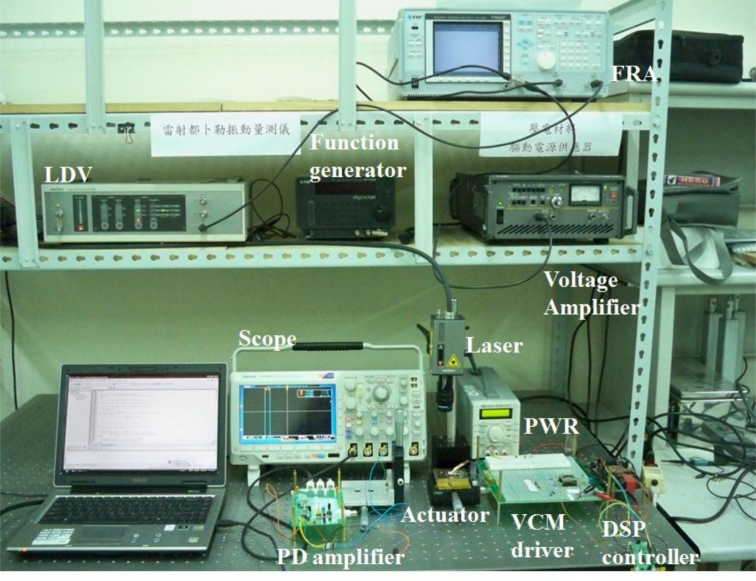
Experimental setup of the dual stage actuator utilized in the active vibration control scheme and measurement point of the laser vibrometer.

Figure 14 shows a prototype of proposed dual-stage PZT-VCM actuator assembly with small passive TVA actuation. The dynamic response of the dual-stage actuator and the PZT bender is obtained experimentally by LDV and a frequency response analyzer, as shown in Figure 15.

**Figure 16.**
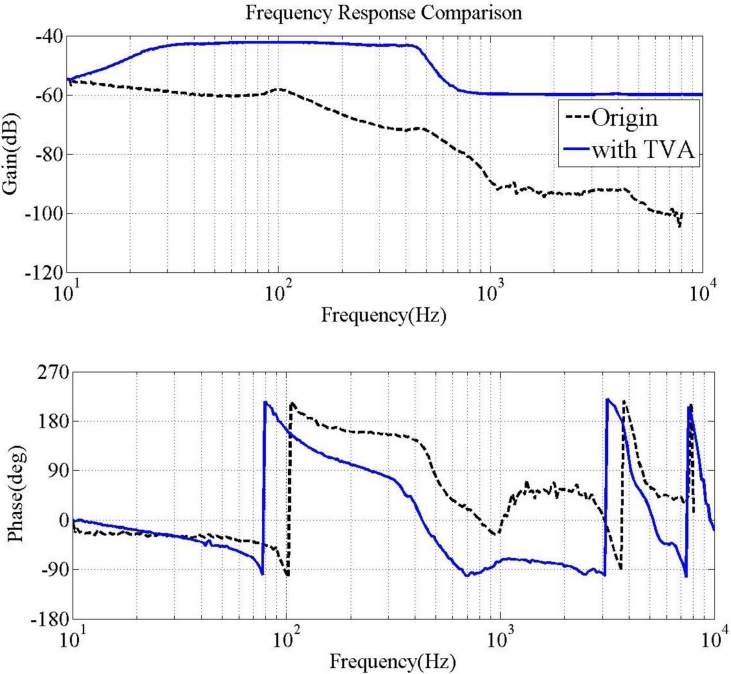
Frequency responses of actuator assemblies with (solid line) and without (dashed line) TVA unit.

Figure 16 shows the effectiveness of TVA vibration suppression. The performance and robustness of the absorber are further enhanced by using optimal processes for dual stage actuation. According to simulation and experimental results, the passive TVA reduces the system vibration by 80% with respect to the peak amplitudes.

**Figure 17.**
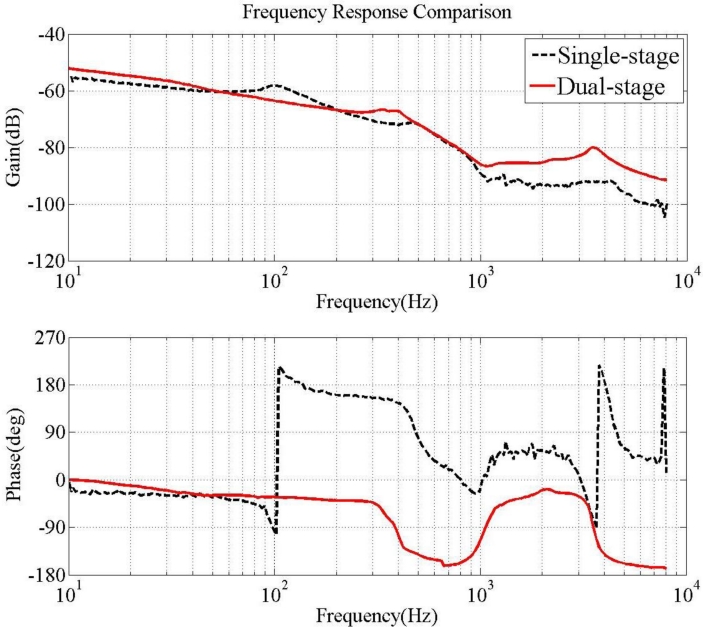
Single- and dual-stage actuated suspension frequency response: VCM input to head displacement (dash line); VCM and PZT combined actuation input to head displacement (solid line).

Design of the PZT loop compensator in the dual-stage actuator expands the bandwidth of the overall system. The primary VCM continuously provides coarse positioning, while the secondary actuator gives fine positioning, vibration and disturbances rejection. Figure 17 illustrates the open loop frequency response of the VCM and PZT dual-stage actuator, compared with the original single-stage VCM actuator.

The open loop bandwidth of the dual-stage system is approximately 3.8 kHz, with a phase margin of 30 degrees and a gain margin of 5 dB. This represents a significant enhancement over the single-stage VCM system, which has a 0.46 kHz bandwidth for the same stability margins. The PZT actuator can reject high frequency disturbances. Notably, the resonant peak of the PZT actuator at 0.9 kHz is lower than the open loop bandwidth and does not pose stability problems.

### 4.2. Tilt Angle Compensation

**Figure 18.**
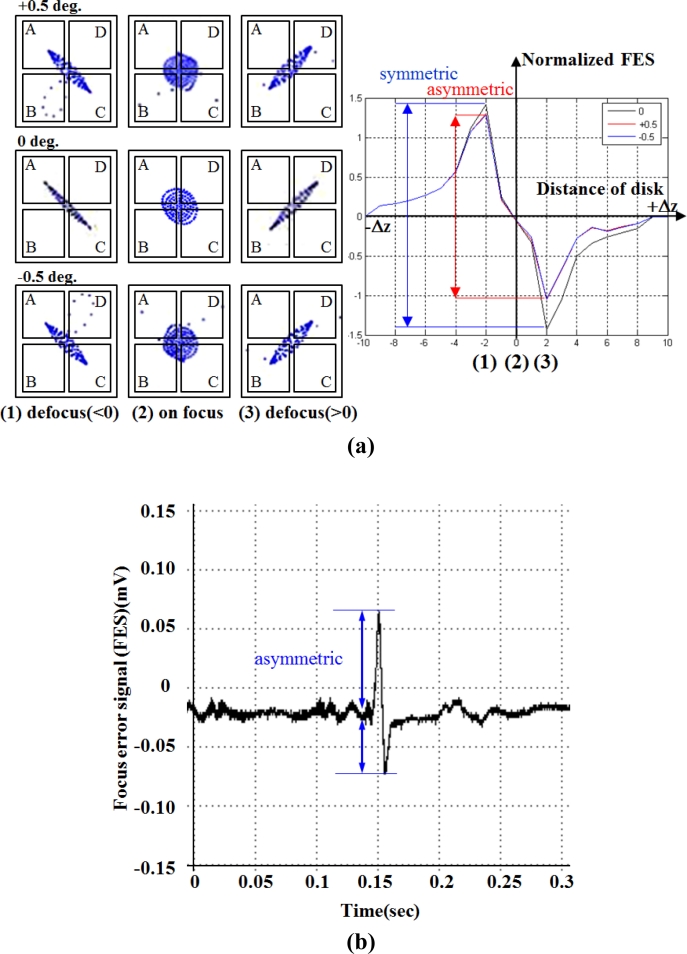
Intensity profiles of the reflected beam on the photo-diode and the output astigmatism signals (S-curve), **(a)** simulation; **(b)** measurement.

**Figure 19.**
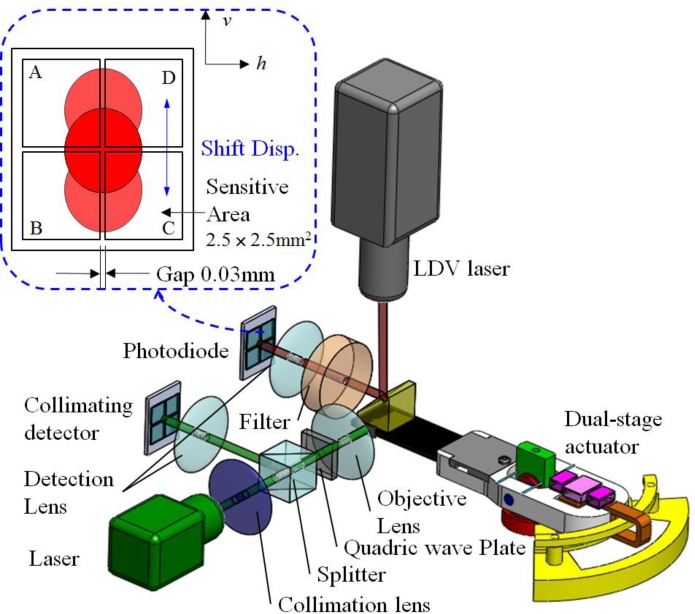
Geometrical model to detect the optical angle: optical layout for the differential detection method and focal spot shifts along the photodiode (*v*-direction).

Figure 18(a) illustrates the simulated focusing error signal (S-curve) generated by the actuator tilting from −0.5^○^ to 0.5^○^, whereas the measured asymmetric S-curve is shown in Figure 18(b) for comparison. The astigmatic returning laser spot deteriorates considerably due to the tilt actuation, causing the asymmetric peak shapes of the S-curve. This unbalanced S-curve signal reduces the accuracy of optical sensing. The optical compensation for dual stage actuator is evaluated by designing the schematic layout of the reflection geometry for tilt measurement [24–26], as shown in Figure 19. The rotary angle measurement is performed by mounting a target 45^°^ micro-prism on the precision VCM-Driven micro-positioning stage. The 635 nm wavelength laser, which is used to adjust tracking angle of dual-stage actuator, produces horizontal beam (green line) and passes through the collimating lens, the polarized beam splitter (PBS), the quarter-wave plate (QWP) to form circular polarization beam and focusing on the side of actuator by objective lens. The returning beam is reflected by the PBS and projected on the collimating detector. Furthermore, the LDV laser beam (red line, vertical) is focusing on the photo-diode to measure the dynamic tilt error motions of focus operation by varying the driving frequency in the range of 5–200 Hz. The distance of the seesaw actuator over the focus actuation stroke is 400 μm or less.

The proposed astigmatic detection method combined with differential phase detection (DPD) is verified through simulation and experimental results. By using the quadrant photo detector array, the astigmatic signal is generated (based on the phase difference between the sum of the diagonal elements, as shown in Equation (22).

Focus error signal (FES), *f*:

(23) $$f\;=\;\frac{\left(A+C)\;-\;(B+D\right)}{\left(A\;+\;B\;+\;C\;+\;D\right)}\times 100\%$$

The DPD signal is defined *versus* the misalignment of the photo-detector in the plane (v, h) by using the following calculation [27,28].

DPD signal, *d_h_*:

(24) $$d_{h}\;=\;\frac{\left(A+B)\;-\;(C+D\right)}{\left(A\;+\;B\;+\;C\;+\;D\right)}\times 100\%$$

DPD signal, *d_v_*:

(25) $$d_{v}\;=\;\frac{\left(A+D)\;-\;(B+C\right)}{\left(A\;+\;B\;+\;C\;+\;D\right)}\times 100\%$$

where *d_h_* and *d_v_* represent the misalignments along the horizontal (*h*) and vertical (*v*) the direction, respectively.

**Figure 20.**
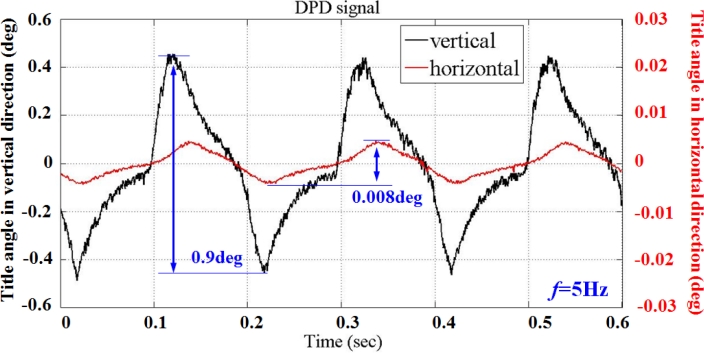
Comparison of two actuation directions with the DPD measurement results from the tilt error signal (black) and lateral deviation (red).

Feasibility of the proposed secondary stage PZT-actuator to compensate for the tilt actuation induced by the primary VCM actuator is demonstrated by conducting an experiment in which the dual stage actuator is kept in the in-focus condition. Figure 20 summarizes the experimental results by moving the prism with reflection geometry. The vertical (black) and horizontal (red) tilt angle are obtained based on differential phase variation with 5 Hz sine-wave excitation. Although the vertical angular displacement involves roll as well as pitch rotation, the phase variation from roll vibration is sufficiently small to be negligible compared with the actuator nutation.

**Figure 21.**
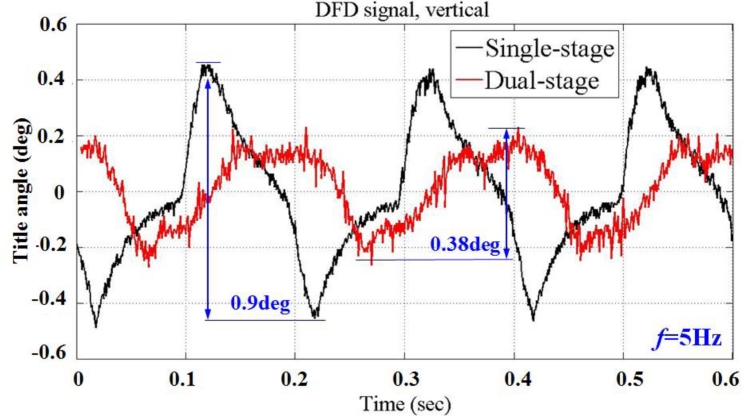
Experimental verification of dual stage compensation: with (red line) and without (black line) piezo-actuated stage actuator.

Both results closely correspond to the numerical calculations. The measurement accuracy of the photo-detector is validated. Figure 21 shows the tilt motions for both single and dual stage control. A sinusoidal signal with a frequency of 5 Hz and voltage amplitude of 6 V is applied to the seesaw actuator. As mentioned earlier, the angular displacement is within the restricted range ±0.19° (peak to peak) for the dual stage actuation than ±0.45° (peak to peak) for single-stage control. The tilt signal variance from a single-stage drive is reduced by 60% with the implementation of this dual stage compensation. Measurement results demonstrate a high stability for the application of adjusting the inclination angle of the optical axis with precision positioning, capable of satisfying the requirement for small tilt angle compensation of a dual stage actuator.

## 5. Conclusions

This work presents a miniaturized seesaw swivel actuator and suspension assembly with a piezoelectric-based micro-actuator and TVA absorber. Among the unique features of the proposed dual stage actuator include a rotary actuator for track-following and a combined piezo-VCM actuated suspension nutation for laser focusing. By using the optimization procedure, the original design is improved in terms of bandwidth and stability. The dynamic response of the dual-stage actuator is also shown, in which the PZT actuator rejects a higher frequency disturbance. The difference between the experimental frequency response of PZT bender and the numerical results is within 5.6%. Lower than the open loop bandwidth, the resonant peak of the PZT-actuator at 0.9 kHz does not pose stability problems. Simulation and experimental results indicate that the passive TVA reduces the system vibration by 80% with respect to the peak amplitudes. This work also investigates the relationship between the tilt sensor sensitivity and working distance of the dual stage actuator based on laser auto-collimation. The tilt variance of optical axis is reduced by 60% from a single-stage drive with the implementation of PZT compensation. Performance optimization of the combined piezo-VCM actuator is also evaluated to demonstrate the effectiveness of the proposed optical sensing applications.

## Figures and Tables

**Table 1.** Parametric optimization investigates the design parameters.

	**Optimization 1**	**Optimization 2**
Objective function	$\begin{array}{lll} E & = & L_{arm}\;(1\;-\;\text{cos}\;\theta )\;+\;L_{\text{PZT}}\;(1\;-\;\text{cos}(\theta \;+\;\phi ))\\ & & -D_{disk}\;(1\;-\;\text{cos}\;\gamma ) \end{array}$	$\omega_{1}\;=\;\sqrt{E_{eq}I_{eq}/m\prime }\times \frac{1.875^{2}}{2\pi L_{\text{PZT}}^{2}}$
Optimization parameter	*L_PZT_*	*t_PZT_*
upper/lower bound	10 ≤*L_arm_* ≤17	10 ≤*L_PZT_* ≤17
	10 ≤*L_PZT_* ≤17	0.1 ≤*t_PZT_* ≤1.0
Constrain	*L_arm_* + *L_PZT_* = 27(mm) *θ* + *ϕ* – *γ* =0(*rad*)	2*t_PZT_* + *t_carbon_* ≤3(*mm*)
Algorithm	*Medium-scale: SQP, Quasi-Newton, line-search*	
Iterations	64	59
Optimization value	**0.271**	**985 Hz**
FuncCount	485	603
Stepsize	0.3014	0.31
Firstorderopt	80.1485	79.3485
Constrviolation	15.0831	21.2495
result	**14.13**	**0.11**

**Table 2.** Verification of numerical optimization.

**Resonant Frequency(**<**10 kHz)**	**Numerical calculation**	**Experimental result**	**Error**

**1st mode**	967	985	1.86%
**2nd mode**	6,065	5,874	3.14%

## Nomenclature

*L_PZT_*: Effective length of PZT (mm)
*L_arm_*: Effective Length of arm (mm)
*D_disc_*: Distance from disk center(mm)
*ϕ*: Rotary angle of PZT bender (rad)
*θ*: Rotary angle of arm (rad)
*γ*: Rotary angle of disk (rad)
*b*: Width of PZT bender (mm)
*t*_PZT_: Thickness of PZT (mm)
*t*_carbon_: Thickness of carbon (mm)
*ρ*_PZT_: Density of PZT (kg/m^3^)
*ρ*_carbon_: Density of carbon (kg/m^3^)
*ρ_abs_*: Density of TVA (kg/m^3^)
*E*_PZT_: Young’s modulus of PZT (GPa)
*E*_carbon_: Young’s modulus of carbon (GPa)
*I*_PZT_: Moment of inertia, PZT (m^4^)
*I*_carbon_: Moment of inertia, carbon (m^4^)
*d_31_*: Charging constant of PZT (C/N)
*V_PZT_*: PZT input Voltage (V)
*A*: The frequency ratio between system and input
*B*: The frequency ratio between system and TVA
*M*: Mass ratio between system and TVA
*m_sys_*: Mass of dual-stage system (kg)
*m_abs_*: Mass of TVA (kg)
*C_PZT_*: Damping constant of PZT
*C_sys_*: Damping constant of arm
*C_abs_*: Damping constant of TVA
*k_sys_*: Stiffness of system (N/m)
*k_PZT_*: Stiffness of PZT (N-m/rad)
*k_eq_*: Equivalent stiffness of PZT (N/m)
*k_abs_*: Stiffness of TVA (N/m)
*ζ_sys_*: Damping ratio of dual-stage system
*ζ_abs_*: Damping ratio of TVA
